# The Extracytoplasmic Domain of the *Mycobacterium tuberculosis* Ser/Thr Kinase PknB Binds Specific Muropeptides and Is Required for PknB Localization

**DOI:** 10.1371/journal.ppat.1002182

**Published:** 2011-07-28

**Authors:** Mushtaq Mir, Jinkeng Asong, Xiuru Li, Jessica Cardot, Geert-Jan Boons, Robert N. Husson

**Affiliations:** 1 Division of Infectious Diseases, Children's Hospital Boston and Harvard Medical School, Boston, Massachusetts, United States of America; 2 Department of Chemistry and the Complex Carbohydrate Research Center, University of Georgia, Athens, Georgia, United States of America; Johns Hopkins School of Medicine, United States of America

## Abstract

The *Mycobacterium tuberculosis* Ser/Thr kinase PknB has been implicated in the regulation of cell growth and morphology in this organism. The extracytoplasmic domain of this membrane protein comprises four penicillin binding protein and Ser/Thr kinase associated (PASTA) domains, which are predicted to bind stem peptides of peptidoglycan. Using a comprehensive library of synthetic muropeptides, we demonstrate that the extracytoplasmic domain of PknB binds muropeptides in a manner dependent on the presence of specific amino acids at the second and third positions of the stem peptide, and on the presence of the sugar moiety N-acetylmuramic acid linked to the peptide. We further show that PknB localizes strongly to the mid-cell and also to the cell poles, and that the extracytoplasmic domain is required for PknB localization. In contrast to strong growth stimulation by conditioned medium, we observe no growth stimulation of *M. tuberculosis* by a synthetic muropeptide with high affinity for the PknB PASTAs. We do find a moderate effect of a high affinity peptide on resuscitation of dormant cells. While the PASTA domains of PknB may play a role in stimulating growth by binding exogenous peptidoglycan fragments, our data indicate that a major function of these domains is for proper PknB localization, likely through binding of peptidoglycan fragments produced locally at the mid-cell and the cell poles. These data suggest a model in which PknB is targeted to the sites of peptidoglycan turnover to regulate cell growth and cell division.

## Author Summary

Regulation of growth by *Mycobacterium tuberculosis* is important in the pathogenesis of tuberculosis (TB), including asymptomatic latent TB infection and active TB disease. The *M. tuberculosis* kinase PknB regulates cell growth and cell division by phosphorylating proteins involved in these processes to modify their function. The activity of PknB is thought to respond to extracellular stimuli by binding specific molecules with its extracytoplasmic domain. In this work we show that cell wall fragments bind to this domain, and that strong binding requires that these interacting molecules have specific molecular features. We demonstrate that a peptidoglycan fragment that binds strongly can stimulate growth of dormant bacteria, but that it does not affect growth of non-dormant bacteria. We also show that PknB localizes to the site of cell division and to the growing tip of the bacterium, where cell wall synthesis and degradation occur, and that the extracytoplasmic domain is required for this localization. These findings indicate that a major function of the extracytoplasmic domain of PknB is to place it at the sites of cell wall turnover, and suggest a model by which PknB can regulate growth and cell division, and thereby contribute to the pathogenesis of TB.

## Introduction

Bacterial cell growth and cell division are highly regulated processes, requiring the coordination of multiple activities within the cell. DNA replication and chromosome segregation for example, must occur at the correct time and in the correct location, and be coordinated with septum formation and cytokinesis. The molecules involved in septum formation and the sequence in which they are recruited to the division site have been the subject of intense investigation in the model organisms *Bacillus subtilis* and *Escherichia coli*, and the identities and functions of many bacterial cell division proteins have been elucidated [1], [2]. In addition to divisome assembly and DNA segregation, bacterial growth and cell division require remodeling of the peptidoglycan (PGN) mesh that forms the cell wall [3]. The enzymes and the sequence of reactions involved in cell wall synthesis are relatively well understood as are the enzymatic activities of many of the PGN hydrolases that can degrade this polymer [4], [5]. In the model organism *B. subtilis*, the mechanisms by which cell wall hydrolases are regulated to achieve morphogenesis are at least partially understood [6]. In other bacteria, including the slow growing actinomycete *Mycobacterium tuberculosis*, less is known about the regulation of PGN synthesis and hydrolysis, how these opposing processes are balanced, and how they are coordinated with other cell processes in growing and dividing vs. non-growing dormant cells.

Because of the apparent ability of *M. tuberculosis* to become dormant in the human host, leading to asymptomatic latent infection, there has been great interest in understanding how cell growth and cell division are regulated in this organism [7]. A longstanding observation that “spent” or “conditioned” medium, i.e. filter-sterilized supernatant from bacterial cultures grown in liquid medium, is able to stimulate growth of dormant cells, led to the identification of a resuscitation promoting factor (Rpf) by purifying from spent medium a component that was able to stimulate growth of the actinomycete *Micrococus luteus* [8]. Rpf is small protein that has homologues in other actinobacteria, including *M. tuberculosis*, which has five *rpf* genes [9]. Functional studies of these genes in *M. tuberculosis* have shown that individually they are not required for resuscitation of dormant *M. tuberculosis* cells and single *rpf* mutant strains do not have other growth or morphologic phenotypes. When two or more *rpf* genes are inactivated, however, growth or resuscitation defects are observed [10], [11], [12]. The recent demonstration that the Rpf's are PGN hydrolases suggests that growth stimulation of dormant cells may result from the enzymatic activity of these secreted proteins, possibly through alterations in PGN structure or through the interaction of PGN degradation products with the bacterial cell surface [13].

A domain found to occur in the extracytoplasmic regions of penicillin binding proteins and serine/threonine kinases (PASTA domain) was identified by bioinformatic analysis and predicted to bind to the stem peptide of un-crosslinked PGN precursors, based on the structure of the PASTA-containing penicillin binding protein PBP2X of *Streptococcus pneumoniae* bound to a cephalosporin antibiotic [14]. Recently the PASTA domain of a Ser/Thr kinase of *B. subtilis* was shown to bind both intact and hydrolyzed PGN [15]. Incubation of *B. subtilis* spores with PGN stimulated spore germination and increased Ser/Thr phosphorylation. Some specificity with respect to the source of PGN and these functional effects was observed, suggesting a preference for meso-diaminopimelic acid (m-DAP)-containing PGN in stimulating spore germination in this organism.

The *M. tuberculosis* genome encodes two proteins that contain PASTA domains, the Ser/Thr protein kinase PknB (Rv0014c) whose extracytoplasmic region comprises four PASTA domains, and the bifunctional penicillin binding protein PBP2 (PonA2, Rv3682), which has a single PASTA domain at the extreme carboxy-terminus of the protein distal to the extracytoplasmic transpeptidase and transglycosylase-containing regions [16]. In this work we investigated the quantitative binding of a series of synthetic muropeptides to the extracytoplasmic region of PknB. We identified specific features of these molecules that are required for high affinity binding, and investigated the functional effects of these compounds *in vivo* on mycobacterial growth, morphology and the localization of PknB. We determined that PknB is strongly localized to septum and less strongly to the cell poles, the sites of active PGN synthesis in mycobacteria, and that the PASTA domains of PknB are required for its localization.

## Results

### Binding of PGN fragments to the extracytoplasmic domain of PknB

The region of *pknB* that encodes the extracytoplasmic domain of PknB (ED-PknB) was amplified by PCR, cloned and ED-PknB was expressed in *Escherichia coli* as an *N*-terminal Glutathione-S-transferase (GST) fusion protein. The ED-PknB comprises 4 PASTA motifs that share limited sequence similarity aside from the key residues that define the motif (Figure S1). Soluble recombinant GST-ED-PknB was affinity purified to >95% purity and after removal of the GST tag was used in subsequent binding experiments (Figure S2).

**Figure 1:**
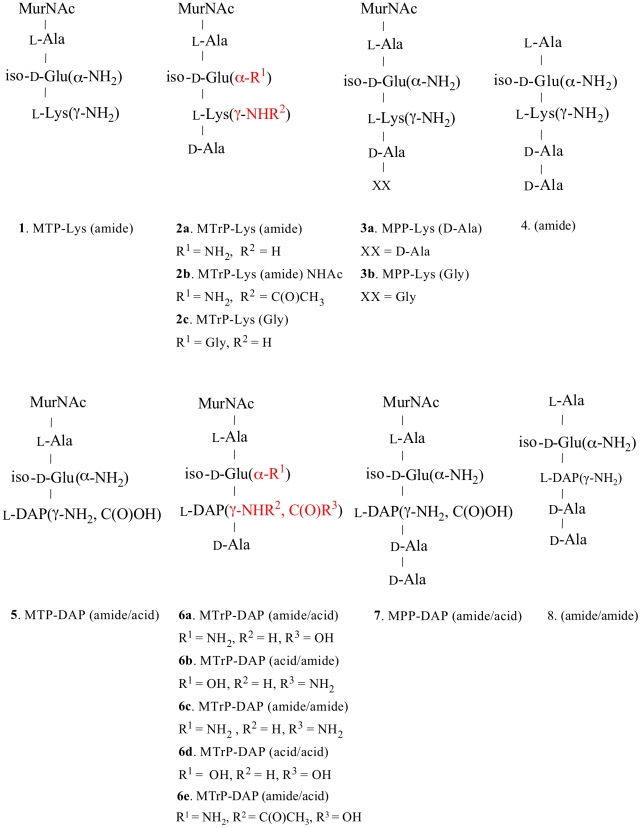
Structures of synthetic muropeptides used in the binding and phenotypic assays. Lys-containing compounds are typical of Gram-positive bacteria and DAP-containing compounds are typical of Gram-negative bacteria and Actinomycetes, including mycobacteria. Variations in substituents are indicated in red, and the specific variations are listed immediately below the structure.

**Figure 2:**
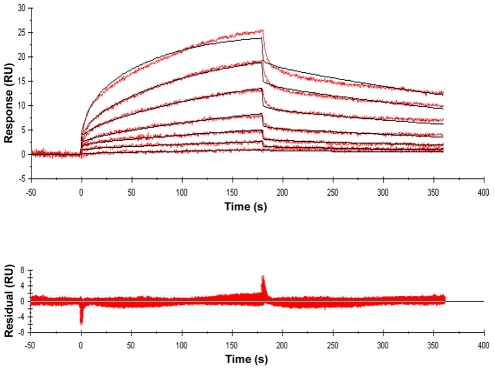
Sensorgrams of a compound with relatively high binding affinity for ED-PknB. The sensorgrams show the simultaneous concentration-dependent kinetic analysis of two-fold serial dilutions of MTrP-DAP (amide/acid) (Compound 6c in Figure 1) at concentrations from 1.56 µM to 100 µM. ED-PknB was bound to the sensor chip and at time 0 the muropeptide was flowed over the chip, followed by a buffer only dissociation step, as described in the Materials and Methods section. Positive deflection of the curve indicates binding in RU (resonance units). The primary data are shown in red. The data were fitted with a two-state binding model (black lines). The corresponding residual values, which are the signal remaining after the data are fitted to the kinetic model, are plotted below the sensorgrams.

A series of PGN fragments (muropeptides) were synthesized as tri-, tetra- and penta-peptides linked to N-acetylmuramic acid (MurNAc) or as unlinked peptides. Amino acids characteristic of PGN stem peptides from Gram-positive bacteria, Gram-negative bacteria or actinobacteria were incorporated into different compounds. Modifications of amino acid side chains that correspond to PGN modifications that are found *in vivo* were also included in the compound series (Figure 1). These compounds were then used in surface plasmon resonance (Biacore) experiments to measure binding affinities of the muropeptides to ED-PknB. To obtain kinetic and thermodynamic parameters, a range of compound concentrations was assayed and kinetic analysis was performed using Biacore Software. An example of a set of sensorgrams for a compound with a relatively low K_D_ is shown in Figure 2. Sensorgrams for the other compounds tested are shown in Figure S3. Table S1 shows detailed kinetic parameters obtained from these experiments.

As shown in Table 1, these experiments demonstrated moderately strong binding of several PGN fragments that have DAP at the third position of the stem peptide. *N*-acetylation of the amino group of DAP as in compound **6e** (MTrP-DAP (amide/acid) NHAc), which is designed to mimic branching of the PGN subunits within the PGN polymeric structure, resulted in a six-fold decrease in binding compared to compound **6a**. The MurNAc-pentapeptide, compound **7**, corresponding to newly synthesized PGN prior to remodeling, bound strongly though about two-fold less than the corresponding MurNAc-tetrapeptide (**6a)**.

**Table 1:** Affinity of synthetic muropeptides for the extracytoplasmic domain of *M. tuberculosis* PknB.

Analyte	K_D_ (µM)
MTP-Lys (amide) **1**	>500
MTrP-Lys (amide) **2a**	21.5
MTrP-Lys (amide) NHAc **2b**	>500
MTrP-Lys (Gly) **2c**	> 500
MPP-Lys (D-Ala) **3a**	> 500
MPP-Lys (Gly) **3b**	>500
Peptide **4** (amide)	> 500
MTP-DAP (amide/acid) **5**	21.8
MTrP-DAP (amide/acid) **6a**	12.7
MTrP-DAP (acid/amide) **6b**	>100
MTrP-DAP (amide/amide) **6c**	14.9
MTrP-DAP (acid/acid) **6d**	53.6
MTrP-DAP(amide/acid)NHAc **6e**	73.8
MPP-DAP (amide/acid) **7**	25.1
Peptide **8** (amide/amide)	>500

Recombinant ED-PknB was immobilized on NHS-activated groups of a CM-5 sensor chip surface (5,000 RU) and titration experiments were performed with the synthetic compounds **1–8** (Figure 1). The binding constants of all compounds were determined by fitting the data using a two-state binding model. Kinetic binding parameters and the sensorgrams for the kinetic analyses are presented in Table S1 and Figure S3. MTP, muramyl-tripeptide; MTrP, muramyl-tetra peptide; MPP, muramyl-pentapeptide.

In addition to preference for DAP at the third position of the stem peptide, another clear result of these experiments is the requirement for amidation of D-isoglutamate (D-iGlu) to D-isoglutamine (D-iGln) at the second position, in order to achieve high affinity binding. Compound **6a**, which contains both D-iGln and DAP at the second and third positions, respectively, exhibited the highest affinity, while compound **6d**, which is identical except for D-iGlu at the second position, bound four-fold less strongly. Similarly, compound **6c**, which also bound with a relatively high affinity (K_D_  = 15 µM), contains D-iGln together with amidation of the carboxyl group of DAP. In contrast, a similar compound, (**6b**), that has D-iGlu at the second position instead of D-iGln did not show measurable binding. The importance of this residue is further underscored by the finding that among the Lys-containing compounds, the only one that showed detectable interaction was compound **2a**, the muramyl tetrapeptide incorporating a D-iGln moiety. While the data indicate a preference for DAP at the third position, the ε-carboxylic acid group that is a major feature that distinguishes DAP from Lys is not an essential requirement for binding. To determine whether the MurNAc moiety was important for binding, compounds **4** and **8**, pentapeptides not linked to MurNAc and containing either Lys or DAP at the third position, respectively, were tested. Neither compound showed significant interaction, indicating an important contribution of MurNAc in binding to the PknB PASTA domains.

### Muropeptides stimulate resuscitation of dormant *M. tuberculosis* cells

The Rpf's have been shown to have PGN hydrolytic activity, and are thought to cleave the ß-1–4 glycosidic linkage between N-acetylmuramic acid and N-acetylglucosamine [13]. This muralytic activity has been shown to be essential for the resuscitation activity of these Rpf proteins, but the mechanism remains uncertain. To determine whether muropeptides that bind to the PASTA domains of ED-PknB can stimulate resuscitation of dormant *M. tuberculosis* cells, we utilized an established *M. tuberculosis* dormancy and resuscitation model [17]. In this assay, *M. tuberculosis* cells are incubated under hypoxic conditions for several months, at which point the number of cells capable of resuming growth in liquid culture is markedly decreased. In this assay, addition of sterile spent medium “resuscitates” dormant cells, leading to an increase in the number of cells that can grow on solid or in liquid medium.

In two independent experiments, we took *M. tuberculosis* stationary phase cultures that had been incubated under hypoxic conditions for 6 or 9 months, and performed this resuscitation assay. In addition to cells incubated in Sauton's medium alone, cells were incubated with a synthetic muropeptide with a high affinity for ED-PknB (**6c** in Figure 1), a muropeptide with low affinity for ED-PknB (**3b** in Figure 1), or with sterile conditioned medium as a positive control. The muropeptides were used at a concentration of 10 times the K_D_ of the high affinity compound as determined in the SPR experiments. Using most probable number analysis [18], which has been used to analyze results from this assay, we observed three and nine-fold increases in the viability of cells that were incubated with the high affinity muropeptide in the two independent experiments. No increase in viability was observed for cells incubated with the low affinity peptide. The cells incubated with sterile spent medium showed a much stronger resuscitation phenotype, with 14 and 100-fold increased viability relative to the cells incubated in fresh medium alone (Table 2).

**Table 2:** Resuscitation of dormant *M. tuberculosis* cultures.

Additive to Culture Medium	Fold increase*
Experiment 1∶6 month old dormant culture	
MTrP-DAP (amide/amide) (6c)	9
MPP-Lys (Gly) (3b)	0.9
50% spent medium	100
Experiment 2∶9 month old dormant culture	
MTrP-DAP (amide/amide) (6c)	3
MPP-Lys (Gly) (3b)	1
50% spent medium	13.6

*Fold increase in viable cell number relative to cultures grown in Sauton's medium without additive.

**Figure 3:**
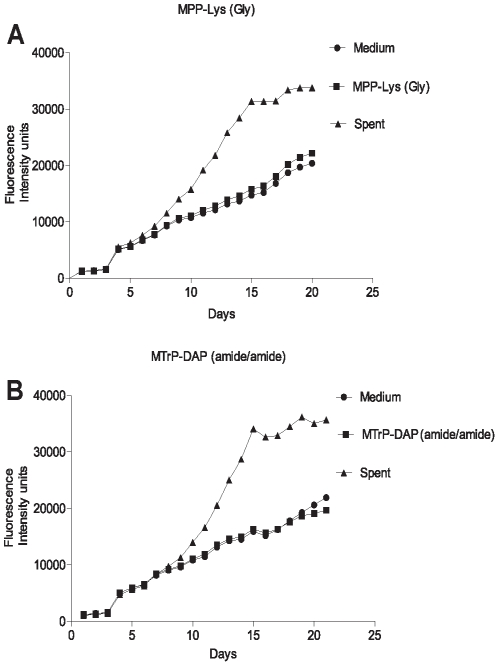
Growth stimulation assay of low inoculum cultures of *M. tuberculosis*. *M. tuberculosis* cells were grown in Sauton's medium alone or in medium supplemented with a synthetic muropeptide at a concentration 10 times the K_D_, of the high affinity muropeptide, or with 50% (v/v) sterile conditioned (spent) medium. Cells were grown to stationary phase, diluted and inoculated into medium containing alamar blue and grown at 37°C, with measurement of fluorescence at 595 nm at serial time points. **A**. Growth curves for MPP-Lys (Compound 3b in Figure 1). **B**. Growth curves for MTrp-DAP (Compound 6c in Figure 1). •, Sauton's medium. ▪, Sauton's medium plus synthetic muropeptide. ▴, Sauton's medium plus spent medium.

The original identification of Rpf in *M. luteus* was based on the observation that stationary phase cells show decreased viability when plated or diluted to low density in liquid medium, but that addition of sterile conditioned medium stimulates growth [8]. A similar phenomenon is observed when mycobacteria are inoculated at low density. To determine whether synthetic muropeptides stimulate growth when stationary phase cells are inoculated at low density, cells from cultures of *M. tuberculosis* (O.D_600_ of 2.4**–**3.6) were washed and diluted 10,000-fold in minimal medium with or without the addition of the high or low affinity muropeptide. As shown in Figure 3, no growth stimulation by either muropeptide was observed in this assay. In contrast, strong growth stimulation by conditioned medium was observed.

### PknB is present in the cell envelope of *M. tuberculosis*

**Figure 4:**
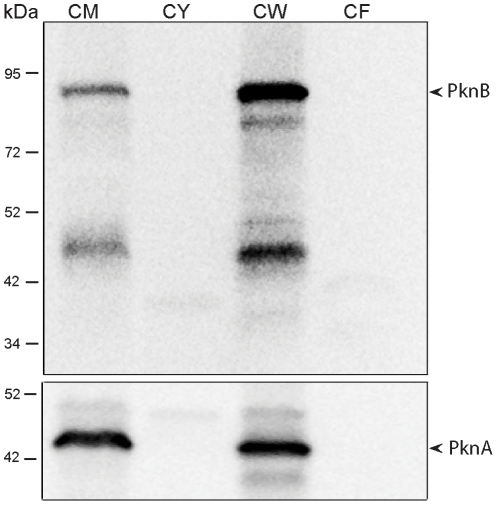
Subcellular localization of *M. tuberculosis* PknB. Top panel: Immunoblot of subcellular fractions of *M. tuberculosis*, probed with a mouse monoclonal antibody that recognizes *M. tuberculosis* ED-PknB. Bottom panel: immunoblot in which the same fractions were probed with rabbit polyclonal sera raised against *M. tuberculosis* membrane protein PknA. CM, membrane fraction; CY, cytoplasmic fraction; CW, cell wall fraction; CF, culture filtrate fraction.

Based on its sequence, PknB is predicted to have a single transmembrane segment, with an intracellular kinase domain and an extracytoplasmic region that incorporates the four PASTA domains [16]. To determine whether PknB is a membrane protein and in which subcellular fraction(s) PknB is located, we performed immunoblotting with a PknB-specific monoclonal antibody. As a control, we probed these subcellular fractions with an antibody to the membrane protein PknA, which like PknB has a single transmembrane segment, but which has a small extracytoplasmic region that is not known to interact with cell wall components. We found that PknB does, as predicted, localize to the membrane fraction of the cell (Figure 4). An even stronger signal was seen in the cell wall fraction, further confirming the association of PknB with the cell envelope. The PknA antibody gave equally strong signals from the membrane and cell wall fractions. These results demonstrate that PknB is a membrane protein and that membrane is present in the cell wall fractions used in these experiments.

### PknB localizes to the septum and poles, and the extracytoplasmic domain is required for proper PknB localization

**Figure 5:**
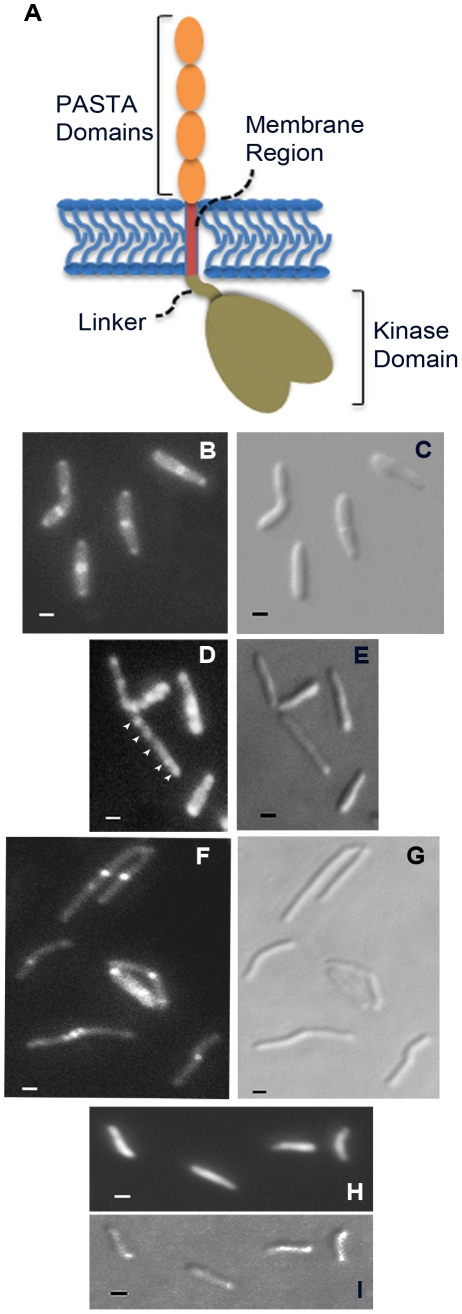
Localization of PknB to sites of peptidoglycan turnover in the mycobacterial cell. A. Schematic representation of domain organization of *M. tuberculosis* PknB, including the kinase domain (residues 1–274; the juxtamembrane linker (residues 275–331), the transmembrane segment (residues 332–354) and the extracytoplasmic PASTA domains (residues 355–626). B–I. Flourescence (B, D, F and H) and DIC (C, E, G and I) images of *M. smegmatis* expressing RFP fused to full-length PknB (B and C), PknB lacking the extracytoplasmic domain (D and E), PknB lacking the intracellular kinase domain and linker (F and G) and unfused RFP (H and I). Arrowheads in panel D point to focal RFP signals along the length of the bacillus. Bar  = 1 µm.

A construct designed to express a PknB-RFP fusion protein, in which RFP is fused to the amino terminus of PknB, was introduced into wild type *M. smegmatis*. Additional constructs, in which RFP is fused a) to the PknB kinase domain, intracellular juxtamembrane sequence and transmembrane segment, but which lacks the extracytoplasmic domain, and b) to the membrane and ED-PknB regions but which lacks the intracellular linker and kinase domains, were also introduced into wild type *M. smegmatis*. Cells were grown to early log phase, expression of the fusion protein was induced, and the cells were examined using fluorescence microscopy (Figure 5). Cells expressing the full-length PknB-RFP fusion showed strong localization of this protein to the mid-cell and symmetrical, less intense localization to both cell poles. In contrast, in cells expressing the fusion that lacks the extracytoplasmic domain containing the PASTA domains, foci of fluorescence were visible at discrete sites along the length of the cell. While in some cells there appears to be increased signal at the poles, we did not observe clear mid-cell localization in cells expressing this construct. To confirm that these foci are not cytoplasmic aggregates, we prepared subcellular fractions of these cells and confirmed that the large majority of this protein is present in the cell membrane and cell wall fractions (Figure S4). Cells expressing the ED-PknB-RFP fusion lacking the intracellular linker and kinase domains showed clear localization to the mid-cell but minimal signal from the poles. This result indicates that the extracytoplasmic PASTA domains are required for proper localization of PknB to the mid-cell and likely to the cell poles and suggests that the intracellular linker-kinase region makes a contribution to localization at the cell poles. To verify these findings, we performed additional imaging of live cells (Figure S5 and Protocol S1), which demonstrates the same localization patterns observed with the fixed cell preparations.

To determine whether diffusible, non-localized muropeptides might bind ED-PknB and disrupt PknB localization, we incubated *M. smegmatis* for 8 hours with the high affinity muropeptide used in the resuscitation experiments. No change in the morphology of wild type bacteria were observed, and in the strain expressing the PknB-RFP fusion protein the RFP signal remained localized to the septum and poles (not shown).

## Discussion

In this work we report three major findings. First, we demonstrated that muropeptides bind to the extracytoplasmic region of PknB, which contains four PASTA domains, and defined molecular requirements for ligand binding. These requirements include both specific residues at the second and third positions in the stem peptide, and the presence of the sugar moiety (MurNAc) linked to the amino-terminal residue of the peptide. Using an extensive series of chemically synthesized compounds, we found moderately high affinity binding by muropeptides that contain DAP at the third position of the stem peptide, in which the D-iGlu at the second position is amidated to D-iGln. The preference for DAP is consistent with the predominant structure of the stem peptide of mycobacteria, where DAP is present at this position, in contrast to most Gram-positive organisms in which Lys occurs at this position. D-iGln at the second position has been reported to be predominant in *M. tuberculosis* PGN, however D-iGlu is present in a minority of stem peptides [19], [20]. Whether synthesis of PGN incorporating D-iGlu vs. D-iGln is site- or growth-stage specific in *M. tuberculosis* is not known. The markedly stronger binding of compounds containing D-iGln suggests that variation in the structure of PGN stem peptides may affect binding by ED-PknB *in vivo*, with potentially important physiologic effects. A recent paper examining stimulation of *B. subtilis* spore germination using synthetic muropeptides confirmed prior results using purified native PGN in showing the importance of DAP at the third position of the stem peptide for this phenotype in this species [21]. In this assay the presence of N-acetylglucosamine linked to MurNAc was also required for potent activity.

A second finding of this work is that PknB localizes strongly to the mid-cell and less strongly to the cell poles of mycobacteria, the sites of active PGN synthesis and hydrolysis in these organisms [22]. Our results with RFP fusions to full-length PknB and to separate domains of this protein in *M. smegmatis* demonstrate that the PASTA motif-containing extracytoplasmic domain of PknB is required for its localization to the mid-cell. We attempted to perform a similar experiment with full-length PknB-RFP in *M. tuberculosis*, however we were unable to obtain consistent expression of the fusion protein. We observed fluorescence in a minority of cells, which was highly variable from cell to cell, and we observed markedly abnormal morphology of many cells, suggesting severe toxicity of *pknB* overexpression, as previously described [23]. Despite these limitations, we were able to see similar localization of full-length PknB in a minority of rod-shaped cells expressing the *pknB-rfp* fusion (data not shown).

In the context of our *in vitro* binding results, these data suggest that binding of PGN fragments by its extracytoplasmic domain is critical for PknB localization to the mid-cell and possibly to the poles. This result, together with the finding that PknB is found in the cell wall and membrane fractions of *M. tuberculosis* lysates, suggests that the PASTA motifs of PknB bind endogenous cell wall or membrane-anchored PGN precursors and/or PGN hydrolysis fragments produced at the septum and poles of the cell. The finding that incubation of growing cells with a high affinity muropeptide had no effect on PknB localization is consistent with this model, and suggests that exogenous muropeptides may not be able to penetrate the complex, lipid-rich mycobacterial cell envelope to reach the PknB PASTA domains at the surface of the cytoplasmic membrane. Because both *de novo* synthesized PGN precursors and PGN hydrolysis products are likely to be localized at the septum and the poles, our data do not indicate which of these are the major PknB PASTA ligands *in vivo*.

The third important finding of this work is that, in contrast to spent medium, which strongly stimulated both growth of non-dormant *M. tuberculosis* cells and resuscitation of dormant cells, a muropeptide with relatively high affinity for the PASTA domains of ED-PknB did not stimulate *M. tuberculosis* growth and had only a modest effect on resuscitation. This result suggests that while muropeptide binding may play a role in resuscitation of *M. tuberculosis,* other factors present in spent medium may be more important in stimulating *M. tuberculosis* growth. In this regard, D-amino acids present in conditioned medium have recently been shown to be a potent growth stimulus for *Vibrio cholerae* and to play a key role in biofilm disruption leading to resumption planktonic growth in *B. subtilis* [24], [25]. Alternatively, PknB may require a different muropeptide ligand, e.g. a disaccharide muropeptide or a multivalent muropeptide, or higher concentrations of these ligands, which may be present *in vivo*, for greater stimulation of growth or resuscitation.

The first structure of a PASTA domain was determined as part of the structure of PBP2x from *S. pneumoniae* bound to a cephalosporin antibiotic [26]. In this structure two PASTA domains interacted to form a compact globular domain. In recent work, the structure of the PASTA motifs of *M. tuberculosis* ED-PknB was determined using NMR and small angle X-ray scattering [27]. While the individual folds of each PASTA domain were similar to those of the PBP2x PASTA domains, the four PASTA domains of PknB are organized as a linear molecule, which is maintained with what the authors termed a ß′/ß′′ brace that prevents interactions between the individual PASTA domains of a single molecule of PknB. A previous structure of the PknB intracellular domain demonstrated the presence of a highly flexible intracellular juxtamembrane segment linking the transmembrane segment to the intracellular kinase domain, indicating that ligand binding resulting in transmembrane propagation of conformational changes leading to PknB activation is unlikely [28].

**Figure 6:**
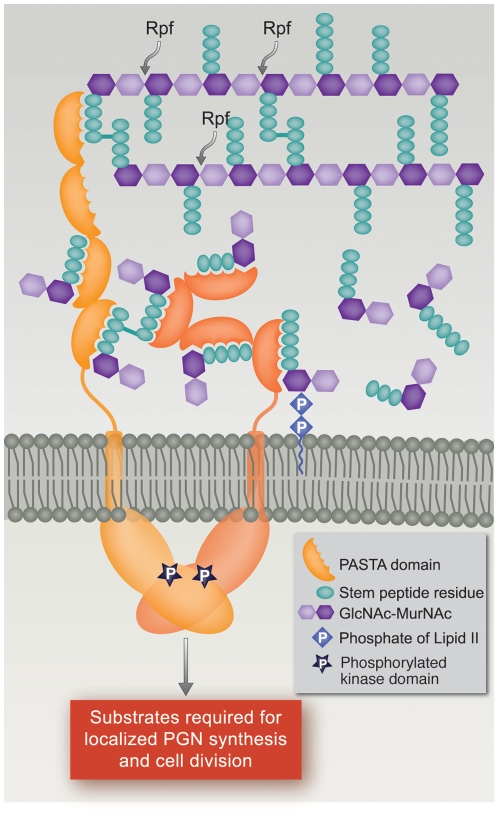
Model of PknB localization and activation by interaction of its extracytoplasmic domain with peptidoglycan fragments. In this model, the extracytoplasmic PASTA domains of PknB bind PGN precursors and/or hydrolysis products produced at the mid-cell and poles, the two sites where active PGN synthesis and degradation occur in mycobacteria. This interaction leads to localization of PknB at the theses sites, and to PknB activation by dimerization of the intracellular kinase domains. Both linear and non-linear forms of the extracytoplasmic domain are shown to allow for possible changes following ligand binding. RPF, resuscitation promoting factor.

Based on the PknB PASTAs structure, a model was proposed in which binding of a single ligand to two molecules of PknB would result in dimerization of the extracytoplasmic domains, which would then cause dimerization of the intracellular kinase domains, resulting in kinase activation [27]. Our data showing relatively high affinity binding of muropeptide monomers, however, suggest an alternative model by which muropeptide binding to ED-PknB could lead to localization and activation of this kinase. In this model, at sites of active PGN hydrolysis and synthesis, i.e. the septum and the cell poles, local concentrations of PGN precursors and PGN hydrolysis products will be high, and binding of these ligands by ED-PknB would result in the septal and polar localization of PknB that we observed. The recruitment of PknB to these sites will consequently result in high concentrations of the intracellular kinase domain, leading to the dimerization that results in kinase activation [28], [29], [30]. PknB activation will then lead to phosphorylation of protein substrates, resulting in regulation of cell division and cell wall synthesis (Figure 6). In this model, there is no requirement for binding of a single muropeptide by PASTA domains from two PknB molecules, or for muropeptides that diffuse from a distance, to achieve PknB localization and activation.

In summary, we have demonstrated sequence-specific binding of muropeptides to the PASTA domain-containing extracytoplasmic region of *M. tuberculosis* PknB, and that the presence of the PASTA domains is required for localization of PknB to sites of PGN turnover. In the context of our phenotypic data and the finding that peptides that bind with high affinity have peptide sequences characteristic of *M. tuberculosis* PGN, our results suggest that in *M. tuberculosis*, the PknB PASTAs bind to PGN precursors or fragments resulting from local PGN synthesis and/or hydrolysis at the mid-cell and poles. This PASTA domain-mediated localization provides a mechanism by which PknB and the co-regulated kinase PknA can regulate cell division and PGN turnover by reversible phosphorylation of proteins involved in these processes, several of which have been shown to be PknA or PknB substrates and to localize to these sites [22], [23], [31], [32], [33].

## Materials and Methods

### Strains, media, and recombinant plasmid construction and protein production

*Escherichia coli* TOP10 (Invitrogen) was used for cloning and was grown in LB broth. *E. coli* BL21 (DE3) (Stratagene) was used for expression of recombinant ED-PknB. *M. tuberculosis* H37Rv or *M. smegmatis* mc^2^-155 were grown at 37°C in Middlebrook 7H9 liquid medium (Difco) supplemented with albumin-dextrose complex (ADC), 0.2% Glycerol and 0.05% Tween 80, except for resuscitation experiments where *M. tuberculosis* was grown in Sauton's medium (Difco). Kanamycin (50 µg ml^−1^) or ampicillin (100 µg ml^−1^) was added to culture media or agar when appropriate.

For expression and purification of ED-PknB, the nucleotide sequence encoding PknB from Gly_354_ to Gln_626_ was PCR-amplified from genomic DNA of *M. tuberculosis* H37Rv and cloned in pGEX-4T-3 (GE Healthcare) for expression as a glutathione-S-transferase (GS) fusion protein. Recombinant GST-ED- PknB was affinity purified to >95% homogeneity using immobilized glutathione agarose (Pierce) (Figure S2). To cleave the GST from the fusion protein, the thrombin CleanCleave kit (Sigma) was used. In brief 900 µg of purified recombinant ED-PknB-GST was incubated with 100 µL of 50% (v/v) suspension of thrombin agarose for 1 hr at room temperature. After centrifugation the supernatant containing ED-PknB and free GST was incubated with 500 µL of 50% (v/v) suspension of Glutathione-agarose for 15 min. After centrifugation the supernatant containing ED-PknB was collected. For SPR analysis the supernatant was dialyzed against phosphate buffered saline (PBS) pH 7.4 prior to use.

For localization of PknB or PknB lacking the extracytoplasmic domain (PknBΔED) in wild *type M. smegmatis*, the full length *pknB* gene or the nucleotide sequence encoding the region from Met_1_ to Gly_354_ of PknB (*pknBΔED*) respectively, were PCR-amplified from genomic DNA of *M. tuberculosis H37Rv*. Overlap PCR amplification of the above PCR products was performed with the PCR product of the red fluorescent protein (*rfp)* gene using a forward primer annealing to the 5′ region of *rfp* and a reverse primer annealing to the 3′ region of *pknB* or *PknBΔED* to obtain the PCR products *rfp-pknB* or *rfp-pknBΔED.* A PacI site was introduced between *rfp* and *pknB.* The fusion PCR products were cloned into the integrating vector pMV306-p_acet_ downstream of the inducible acetamide promoter at NdeI and XbaI sites to obtain pMV306-p_acet_-*rfp-pknB* or pMV306-p_acet_-*rfp- pknBΔED*. To obtain recombinant clone pMV306-p_acet_-*rfp-pknBΔKD* expressing RFP linked to transmembrane and extracellular domains of PknB (ED-PknB-RFP) the *pknB* gene of the clone pMV306-p_acet_-*rfp-pknB* was replaced with the nucleotide sequence encoding Ile_326_ - Gln_626_ using PacI and XbaI sites. Cloned DNA was sequenced to verify the absence of mutations. A mycobacterial replicating vector that constitutively expresses RFP was a gift from Eric Rubin.

### Chemical synthesis of PGN fragments

Compounds were synthesized using classical fluorenylmethoxycarbonyl (Fmoc) chemistry and standard manual solid-phase peptide synthesis techniques as previously described [34], [35], [36], [37], [38], [39]. In the preparation of the peptide portion of the compounds, Sieber Amide resin was swelled in dimethylformamide (DMF) for 45 min and then treated with piperidine in DMF. After a reaction time of 30 min, the solvents were removed by filtration and the resin washed with DMF, and then treated with Fmoc-linked amino acid building blocks. This generated PGN partial structures with N-termini.

Compound 8 was newly synthesized for this work as follows. Rink amide AM LL resin (0.1 mmol) was swelled in dichloromethane for 30 min and then rinsed with DMF (3×5 mL). The resin was treated with piperidine in DMF (20%, 3×5 mL). After a reaction time of 30 min, the solvents were removed by filtration and the resin was washed with DMF (3×5 mL), followed by treatment with Fmoc-D-Ala-OH (155.7 mg, 0.5 mmol) in DMF in the presence of HATU (mg, 0.5 mmol) and DIPEA (mL). The reaction progress was monitored by a Kaiser test. After completion of the coupling, the resin was washed with DMF (3×5 mL). The Fmoc protecting group was removed by treatment with piperidine in DMF (20%, 3×5 mL, 3×10 min). The reaction cycle was repeated using Fmoc-D-Ala-OH (155.7 mg, 0.5 mmol), Fmoc-DAP(BOC,tBu)-OH (113.7 mg, 0.2 mmol), Fmoc-D-iso-Gln-OH (73.7 mg, 0.2 mmol), Fmoc-L-Ala-OH (155.7 mg, 0.5 mmol). The final Fmoc protecting group was removed by treatment with piperidine in DMF (20%, 3×5 mL, 3×10 min). The resin was washed with DMF (3×5 mL) and the resin bound peptide was capped by treatment with acetic anhydride and (10%) and DIPEA (5%) in DMF (3×5 mL, 3×10 min). The resin was washed with DMF (3×5 mL), dichloromethane (7×5 mL), and methanol (3×5 mL) and dried *in vacuo* overnight. The peptide was released from the resin by treatment with TFA/TIPS/Water (95%/2.5%/2.5%) in DMF for 2 h. The resin was filtered and washed with TFA (1×10 mL). The filtrate was concentrated under reduced pressure and co-evaporated with toluene. The crude peptide was dissolved in water/acetonitrile and purified by semi-preparative HPLC (Eclipse XDB-C18 column, 5 mm, 9.4×250 mm, eluent: water/acetonitrile/0.1%TFA) to afford, after lyophilization of the appropriate fraction, the target compound **8**. HRMS-MALDI-TOF calculated for C_23_H_40_N_8_O_9_Na [M + Na]: 595.29, experimental: 595.41.

### Surface plasmon resonance binding assays and kinetic analysis

Binding interactions between ED-PknB and PGN analytes were examined using a Biacore T100 biosensor system (Biacore Life Sciences - GE Healthcare). Soluble ED-PknB was immobilized by standard amine coupling using an amine coupling kit (Biacore). The surface was activated using freshly mixed N-hydroxysuccimide (NHS; 100 mM) and 1-(3-dimethylaminopropyl)-ethylcarbodiimide (EDC; 391 mM) (1/1, v/v) in water. Next, ED-PknB (50 µg/ml) in aqueous NaOAc (10 mM, pH 4.5) was passed over the chip surface until a ligand density of approximately 5,000 RU was achieved. The remaining active esters were quenched by aqueous ethanolamine (1.0 M; pH 8.5). The control flow cell was activated with NHS and EDC followed by immediate quenching with ethanolamine. HBS-EP (0.01 M HEPES, 150 mM NaCl, 3 mM EDTA, 0.005% polysorbate 20; pH 7.4) was used as the running buffer for the immobilization and kinetic studies. Analytes were dissolved in running buffer and a flow rate of 20 µL/min was employed for association and dissociation at a constant temperature of 25°C. A double sequential 60 s injection of aqueous NaOH (50 mM; pH 11.0) at a flow rate of 50 µl/min followed by 5 min stabilization with running buffer was used for regeneration and achieved prior baseline status. The same experimental surface was used for approximately 4 weeks and maintained under running buffer conditions. MTP-DAP (amide/acid) (**5**) was used as a positive control in each experiment to check the stability of the ED-PknB surface activity during the course of the experiments.

To minimize bulk refractive index changes, nonspecific binding and instrument drift on the generated binding sensorgrams, a double referencing of the data was performed. First, bulk refractive index change effects were minimized by preparing all analytes in the HBS-EP buffer. Then, the binding responses over the reference surface were subtracted from the active surface to correct for nonspecific binding. A blank analyte run of running buffer alone was also subtracted from the specific binding sensorgrams to minimize instrument noise. Using Biacore T100 evaluation software, the response curves of various analyte concentrations were globally fitted to the two-state binding model [40].

### Conditioned medium preparation

Conditioned medium was prepared as previously described [41]. Briefly, supernatant was obtained from *M. tuberculosis* H37Rv culture grown in ADC-supplemented Sauton's medium containing 0.05% Tween 80 at 37°C with shaking to an optical density at 600 nm (OD_600_) of 1.2. After centrifugation (4000 rpm, 10 min), the supernatant was sterilized by passage through 0.22 µm filter, tested for sterility, and used for the resuscitation experiments.

### Dormancy and resuscitation assay

To obtain non-culturable dormant bacilli, *Mycobacterium tuberculosis* H37Rv was grown under long-term oxygen starvation in broth growth medium (Sauton's medium containing 0.05% tween 80 and supplemented with ADC) as previously described [10]. In brief, *M. tuberculosis* was initially grown to late stationary phase at 37°C with shaking. From this initial culture, 100 µL was subcultured into 20 ml of growth medium and grown to an optical density at 600 nm (OD_600)_ of 1.8 to 2. Finally 100 µL was inoculated into 75 ml of growth medium containing 1.5 µg/ml methylene blue in a sealed 250-ml flask and grown with shaking at 37°C for 6 or 9 months. Methylene blue became colorless by 10 days of incubation, indicating oxygen depletion.

For resuscitation experiments muropeptides were dissolved in sterile Sauton's medium to a concentration of 20 times the binding constant (K_D_) of the high affinity compound (**6c**) The dormant culture was serially diluted (10^−1^ to 10^−6^) in growth medium. From each dilution 4 sets of triplicate 100 µL culture were aliquoted in wells of 96 well plates (one set each for muropeptides (2 muropeptides tested), growth medium and spent medium). 100 µL of growth medium, muropeptide, or spent medium was added to each well of the corresponding set. The final concentration of muropeptide was 10 times the K_D_ of the high affinity compound, and of the spent medium was 50%. Plates were incubated at 37°C. Drying was prevented by maintaining sterile water in outer wells of the plate. After 2 months the wells with visibly turbid growth were recorded and MPN values were calculated as previously described [42].

### Growth stimulation assay

To investigate the effect of muropeptides on growth initiation of low inoculum cultures of *M. tuberculosis*, stationary phase (O.D_600_ of 2.4–3.6) cultures grown in Sauton's medium supplemented with ADC and 0.05% Tween 80 were passed through five micron pore filter (Millipore) to remove clumps. To obtain a single cell suspension, the culture was passed five times through a 27½ gauge needle followed by washing three times with Sauton's medium. 100 µl of a 10^−4^ dilution was inoculated into wells of a 96 well plate for a final volume of 200 µl. 1x alamar Blue was included in each well. As above, the final concentration of the muropeptides was 10 times the K_D_ of the high affinity compound, and of the spent medium was 50%. Each condition was tested in duplicate. Growth was monitored in each well by measuring fluorescence using excitation of 550 nm and emission of 595 nm and plotted as fluorescence intensity units versus time in days.

### Microscopy

For cellular localization of RFP fusions to intact PknB or its domains, the corresponding plasmid expressing the fusion under control of the acetamidase promoter was electroporated into *M. smegmatis* cells. The resulting strains were grown in Middlebrook 7H9 liquid medium supplemented with ADC, 0.2% Glycerol and 0.05% Tween 80 to mid-log phase, followed by induction with 0.2% acetamide for 6 hrs. Cells were fixed in 4% paraformaldehyde at 37°C for 30 min followed by incubation with 50 mM ammonium chloride for 5 min at room temperature. Cells were transferred onto a glass slide, air-dried and one drop of Prolong Gold antifade reagent (Invitrogen) was applied before covering with a coverslip. After 24 hrs of curing in the dark, cells were observed using a Zeiss Axiophot microscope with a 63x differential interference contrast (DIC) oil immersion objective and red fluorescence filter. Images were captured by a Spot cooled CCD camera (Diagnostic Instruments), acquired with Spot software and processed by Adobe Photoshop CS2.

### Western blotting

For cellular localization of native PknB in wild type *M. tuberculosis* cells, 60 µg total protein of cytosol, cell wall, cell membrane and culture filtrate fractions, prepared at Colorado Statue University and obtained from the Biodefense and Emerging Infections Research Resources Repository, was fractionated on 10% SDS-PAGE and transferred to a PVDF membrane. The blot was blocked in Tris-buffered saline containing 0.1% Tween 20 (TBST) and 5% milk for 1 hr at room temperature. The blot was incubated overnight at 4°C with 1∶10,000 dilutions of either a mouse monoclonal antibody raised against extracytoplasmic domain of PknB or with a rabbit polyclonal antibody against PknA. After thorough washing with TBST, the blot was incubated with a 1∶10,000 dilution of HRP-conjugated secondary antibodies (Cell Signaling) for 3 hrs at room temperature. Finally after 3 washes with TBST the blot was developed with Lumiglo (Cell Signaling) and the blot image was obtained on a Kodak Image Station 4000.

### Accession numbers

PknA: P65726

PknB: POA5S4

## Supporting Information

Figure S1 **T-Coffee alignment of the four PASTA domains of** ***M. tuberculosis*** **PknB and the single PASTA domain of PBP2.** Residues/positions corresponding to those that are conserved in PASTA domains from multiple bacterial species, according to reference 14, are highlighted in blue.
(TIF)

Figure S2 **SDS-PAGE gel showing expression and purification of ED-PknB.** M, molecular weight markers: UI, lysate from uninduced cultures; I, lysate from induced cultures, The purified protein following removal of the GST tag, shown in the last lane on the right, was used in the binding experiments.
(TIF)

Figure S3 **Sensorgrams of compounds tested in the Biacore binding experiments.** The sensorgrams show the simultaneous concentration-dependent kinetic analysis of two-fold serial dilutions of each compound. ED-PknB was bound to the sensor chip and at time 0 the muropeptide was flowed over the chip as described in the Materials and Methods section. Positive deflection of the curve indicates binding in RU (resonance units). The primary data are shown in red. For compounds that showed significant binding the data were fitted with a two-state binding model (black lines) and the corresponding residual values, which are the signal remaining after the data are fitted to the kinetic model, are plotted below the sensorgrams. Sensorgrams for individual muropeptides are shown in A) MTP-Lys (amide) (1); B) MTrP-Lys (amide) (2a); C) MTrP-Lys (amide) NHAc (2b); D) MTrP-Lys (Gly) (2c); E) MPP-Lys (D-Ala) (3a); F) MPP-Lys (Gly) (3b); G) Peptide (amide) (4); H) MTP-DAP (amide/acid) (5); I) MTrP-DAP (amide/acid) (6a); J) MTrP-DAP (acid/amide) (6b); K) MTrP-DAP (acid/acid) (6d); L) MTrP-DAP(amide/acid)NHAc (6e); M) MPP-DAP (amide/acid) (7); N) Peptide (amide/amide) (8).
(PDF)

Figure S4 **Immunoblot of subcellular fractions of** ***M. smegmatis*** **showing localization of the RFP-PknB kinase domain fusion.** *M. smegmatis* was grown to mid log phase, acetamide was added at a concentration of 0.2% for 8 hours. Cells were harvested, lysed with a French Press and sub-cellular fractions were isolated using the protocol developed by the TB Research Materials Contract at Colorado State University (http://www.cvmbs.colostate.edu/mip/tb/pdf/scf.pdf). RH615, *M. smegmatis* expressing the RFP-PknB kinase domain fusion under control of the inducible acetamidase promoter; CM, cytoplasmic membrane fraction; CY, cytoplasm; CW, Cell wall fraction. Though some fusion protein is present in the cytoplasm, the majority is in the cell wall and cell membrane fractions, as is native *M. smegmatis* PknB.
(TIF)

Figure S5 **Live cell imaging of** ***M. smegmatis*** **.** Cells expressing RFP fused to a) full-length PknB, b) to the kinase domain, linker and transmembrane segment, or c) to ED-PknB and the transmembrane segment. Fluorescence images are shown on the left and DIC images on the right. Bar  = 1 µm.
(TIF)

Table S1 Kinetic binding parameters for the interaction of synthetic muropeptides with the PASTA domains of *M. tuberculosis* PknB.
(DOC)

Protocol S1 Methods for live cell imaging of *M. smegmatis* expressing RFP-PknB fusions.
(DOCX)
