# Supplementary figures and images for: The Extracytoplasmic Domain of the Mycobacterium tuberculosis Ser/Thr Kinase PknB Binds Specific Muropeptides and Is Required for PknB Localization
Source: PLoS Pathog. 2011 Jul 28;7(7):e1002182. doi: 10.1371/journal.ppat.1002182 (PMC3145798; doi:10.1371/journal.ppat.1002182)

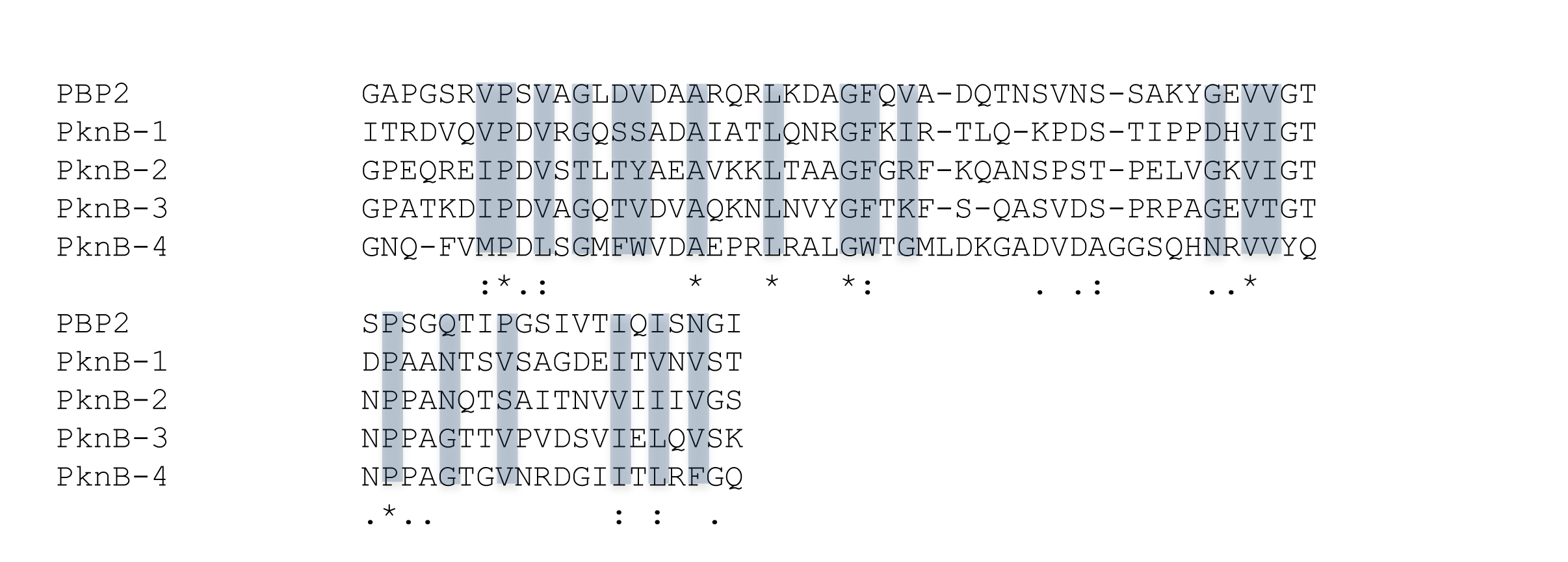

Supplement: Figure S1 — T-Coffee alignment of the four PASTA domains of M. tuberculosis PknB and the single PASTA domain of PBP2. Residues/positions corresponding to those that are conserved in PASTA domains from multiple bacterial species, according to reference 14, are highlighted in blue. (TIF) [file ppat.1002182.s001.tif]

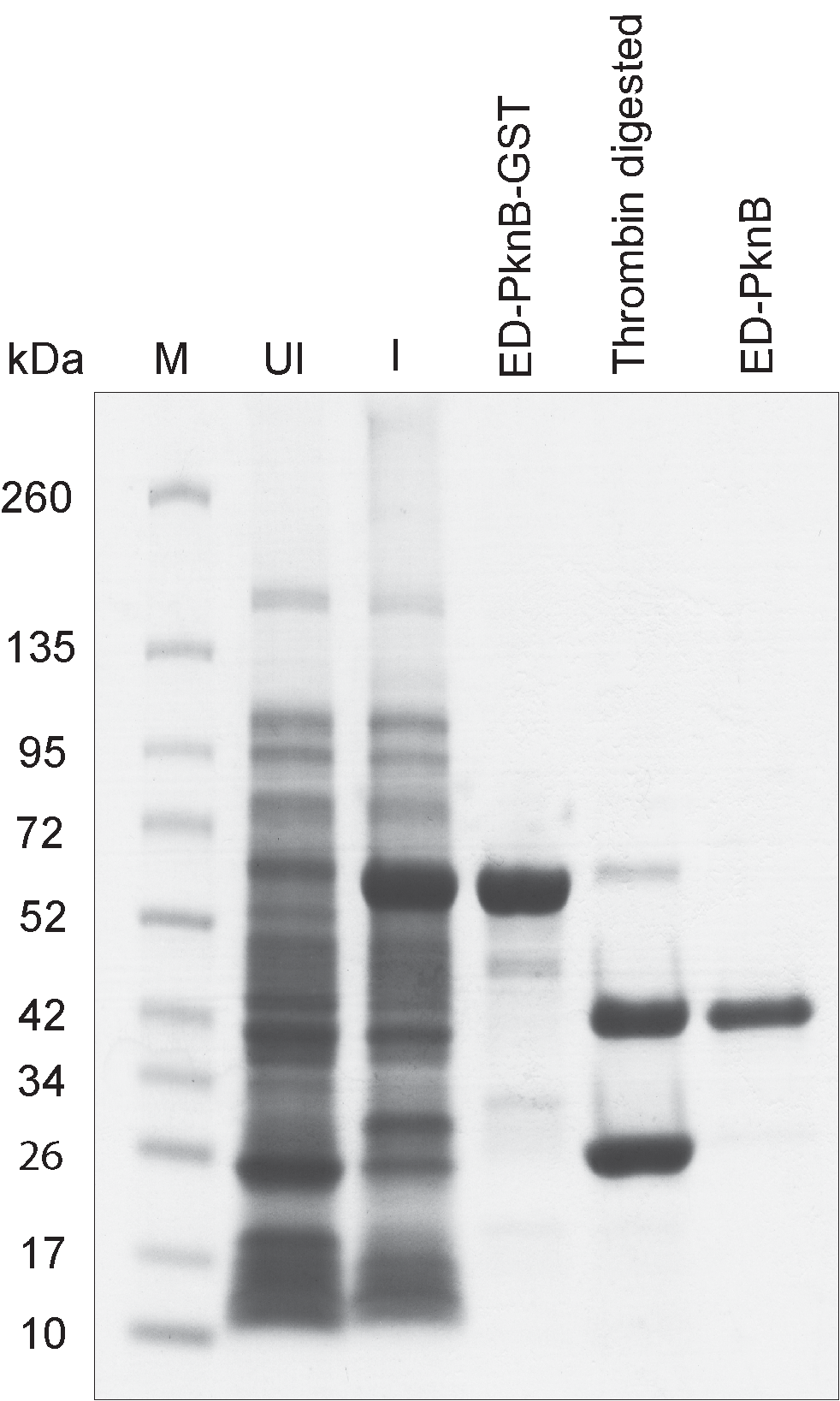

Supplement: Figure S2 — SDS-PAGE gel showing expression and purification of ED-PknB. M, molecular weight markers: UI, lysate from uninduced cultures; I, lysate from induced cultures, The purified protein following removal of the GST tag, shown in the last lane on the right, was used in the binding experiments. (TIF) [file ppat.1002182.s002.tif]

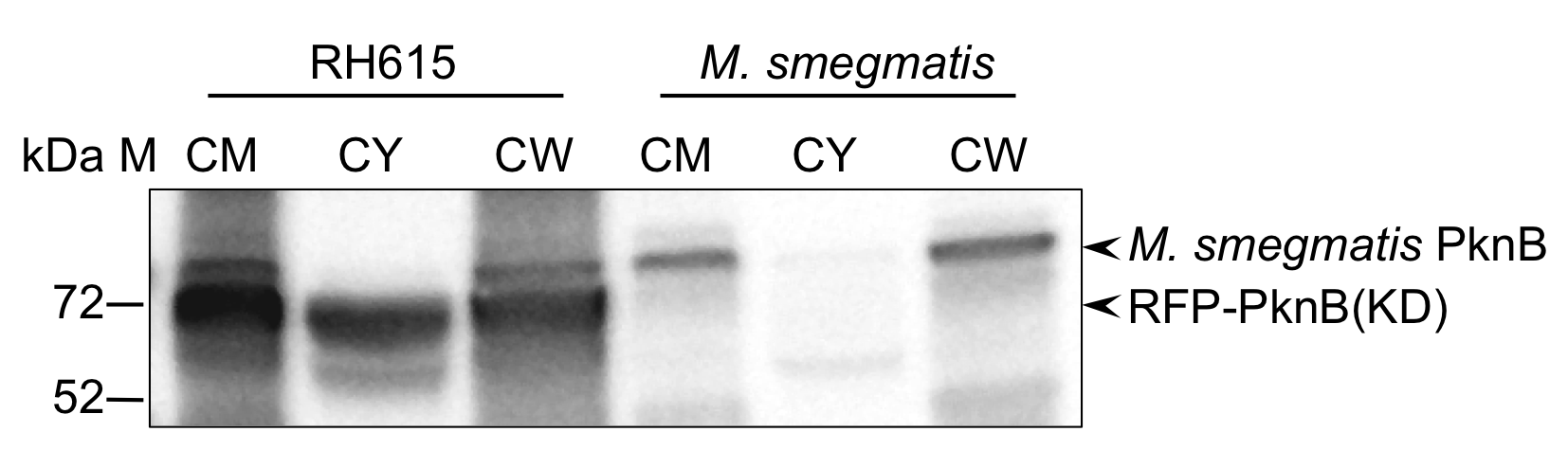

Supplement: Figure S4 — Immunoblot of subcellular fractions of M. smegmatis showing localization of the RFP-PknB kinase domain fusion. M. smegmatis was grown to mid log phase, acetamide was added at a concentration of 0.2% for 8 hours. Cells were harvested, lysed with a French Press and sub-cellular fractions were isolated using the protocol developed by the TB Research Materials Contract at Colorado State University (http://www.cvmbs.colostate.edu/mip/tb/pdf/scf.pdf). RH615, M. smegmatis expressing the RFP-PknB kinase domain fusion under control of the inducible acetamidase promoter; CM, cytoplasmic membrane fraction; CY, cytoplasm; CW, Cell wall fraction. Though some fusion protein is present in the cytoplasm, the majority is in the cell wall and cell membrane fractions, as is native M. smegmatis PknB. (TIF) [file ppat.1002182.s004.tif]

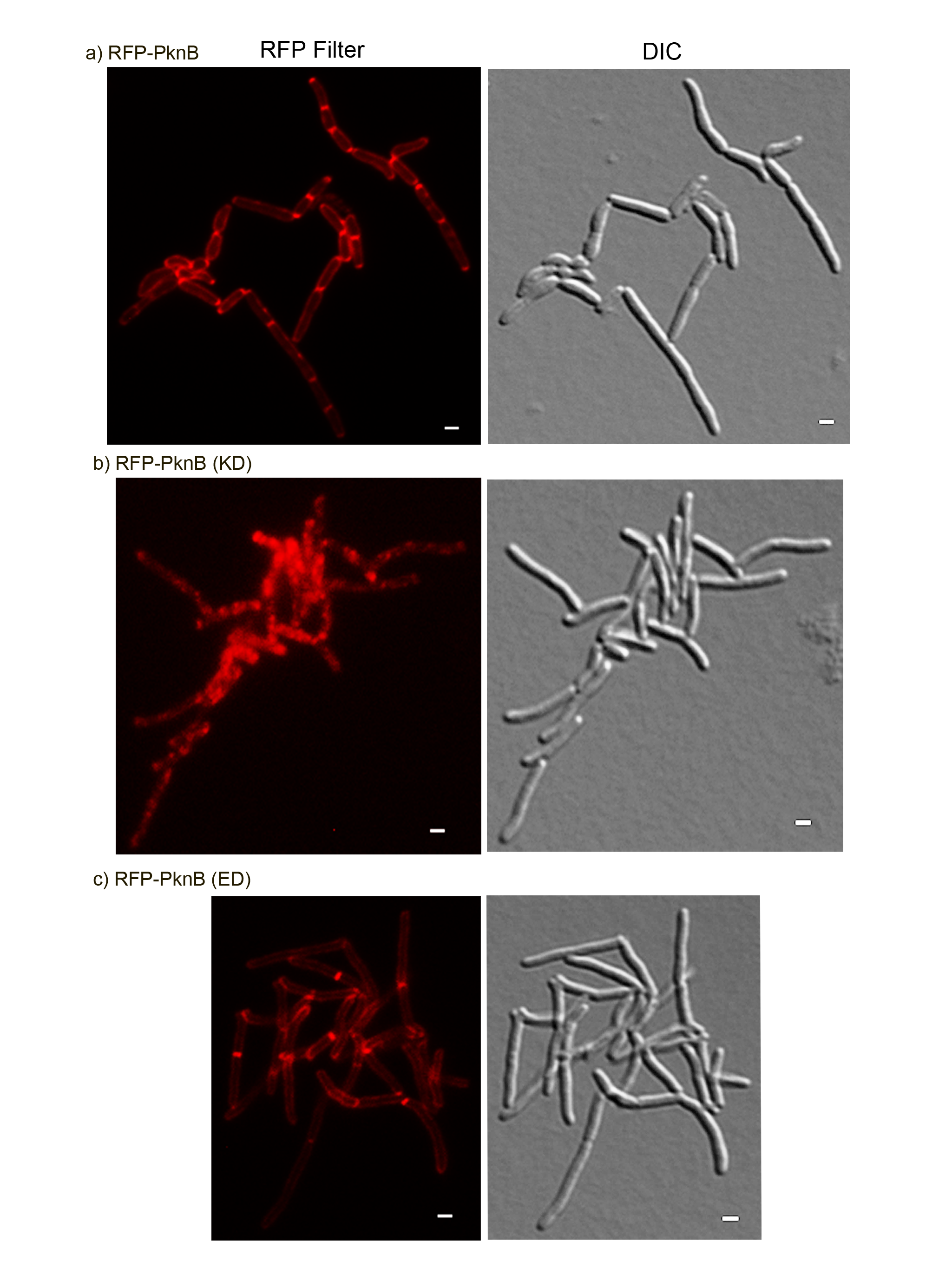

Supplement: Figure S5 — Live cell imaging of M. smegmatis . Cells expressing RFP fused to a) full-length PknB, b) to the kinase domain, linker and transmembrane segment, or c) to ED-PknB and the transmembrane segment. Fluorescence images are shown on the left and DIC images on the right. Bar = 1 µm. (TIF) [file ppat.1002182.s005.tif]
