# Supplementary material for: The Extracytoplasmic Domain of the Mycobacterium tuberculosis Ser/Thr Kinase PknB Binds Specific Muropeptides and Is Required for PknB Localization
Source: PLoS Pathog. 2011 Jul 28;7(7):e1002182. doi: 10.1371/journal.ppat.1002182 (PMC3145798; doi:10.1371/journal.ppat.1002182)
Supplement: Figure S3 — Sensorgrams of compounds tested in the Biacore binding experiments. The sensorgrams show the simultaneous concentration-dependent kinetic analysis of two-fold serial dilutions of each compound. ED-PknB was bound to the sensor chip and at time 0 the muropeptide was flowed over the chip as described in the Materials and Methods section. Positive deflection of the curve indicates binding in RU (resonance units). The primary data are shown in red. For compounds that showed significant binding the data were fitted with a two-state binding model (black lines) and the corresponding residual values, which are the signal remaining after the data are fitted to the kinetic model, are plotted below the sensorgrams. Sensorgrams for individual muropeptides are shown in A) MTP-Lys (amide) (1); B) MTrP-Lys (amide) (2a); C) MTrP-Lys (amide) NHAc (2b); D) MTrP-Lys (Gly) (2c); E) MPP-Lys (D-Ala) (3a); F) MPP-Lys (Gly) (3b); G) Peptide (amide) (4); H) MTP-DAP (amide/acid) (5); I) MTrP-DAP (amide/acid) (6a); J) MTrP-DAP (acid/amide) (6b); K) MTrP-DAP (acid/acid) (6d); L) MTrP-DAP(amide/acid)NHAc (6e); M) MPP-DAP (amide/acid) (7); N) Peptide (amide/amide) (8). (PDF) [file ppat.1002182.s003.pdf]

Sensorgrams of Lysine containing muropeptides

A) MTP-Lys (amide) (**1**)

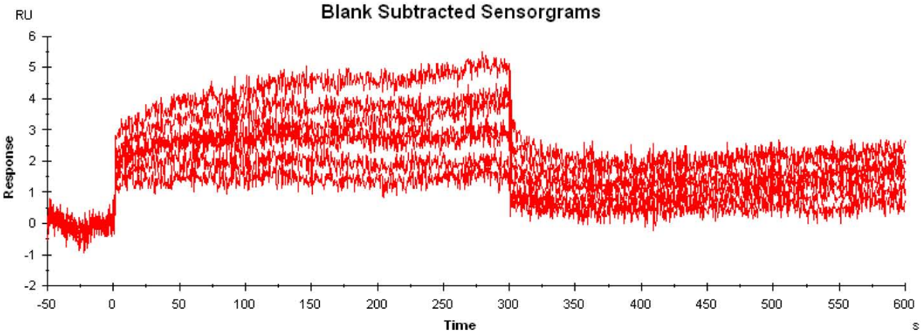

B) MTrP-Lys (amide) (**2a**)

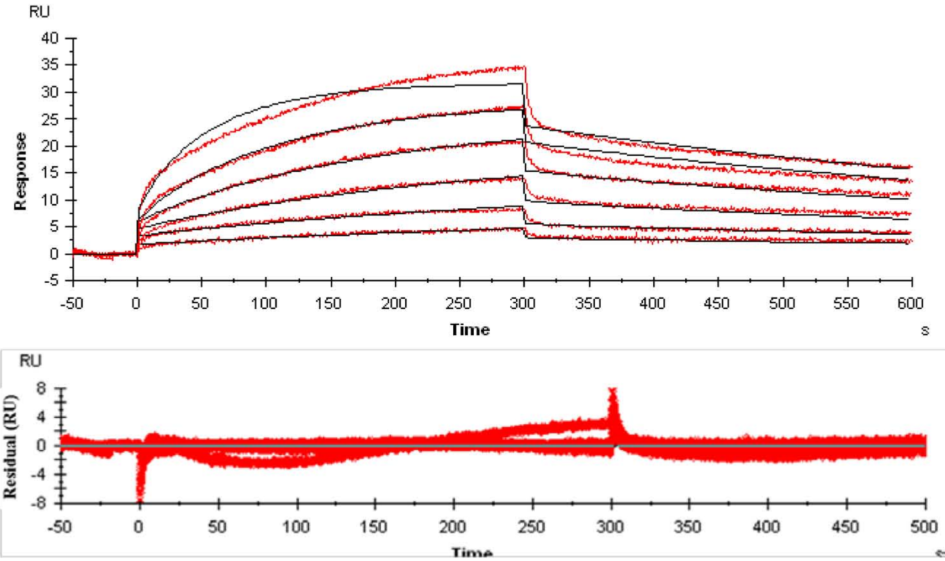

C) MTrP-Lys (amide) NHAc **(2b)**

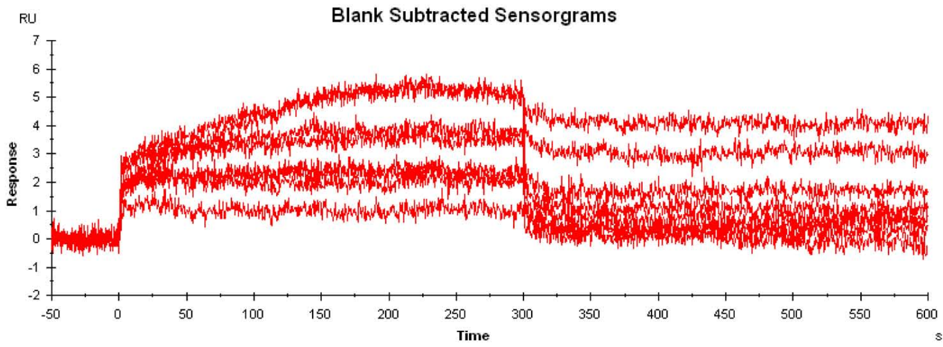

D) MTrP-Lys (Gly) (**2c)**

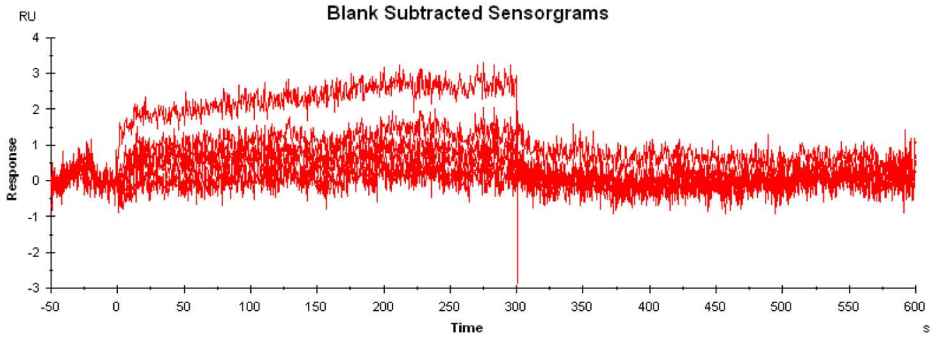

E) MPP-Lys (D-Ala) (**3a)**

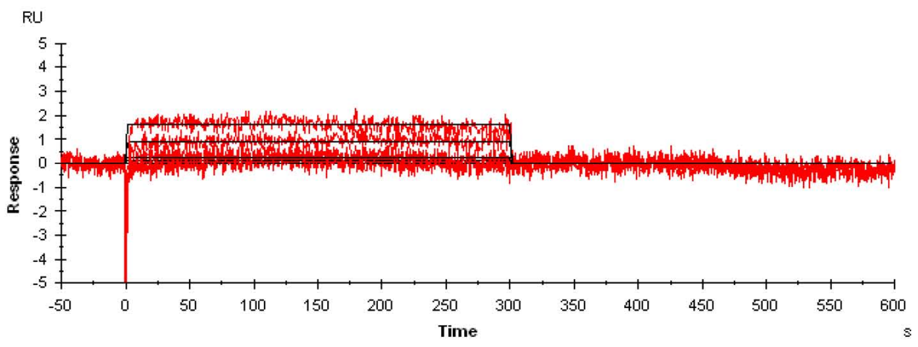

F) MPP-Lys (Gly) (**3b**)

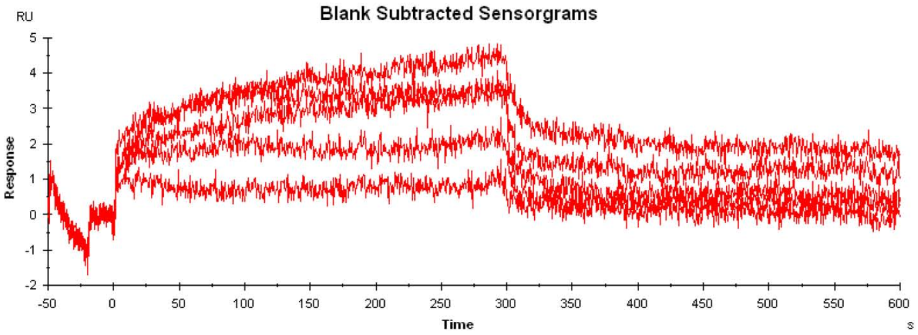

G) Peptide (**4**)

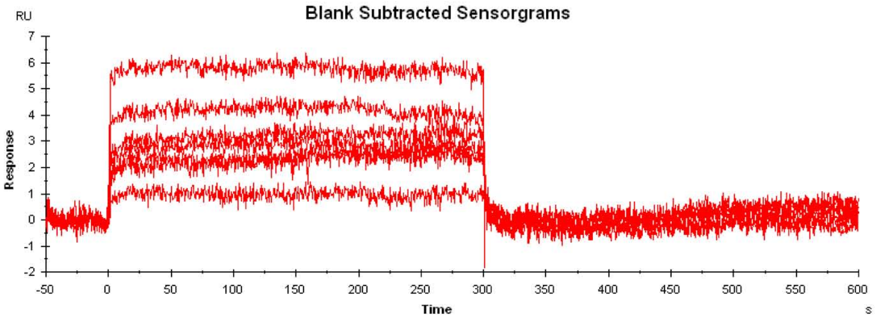

Sensorgrams of DAP Containing Peptidoglycan part structures

H) MTP-DAP (amide/acid) (**5**)

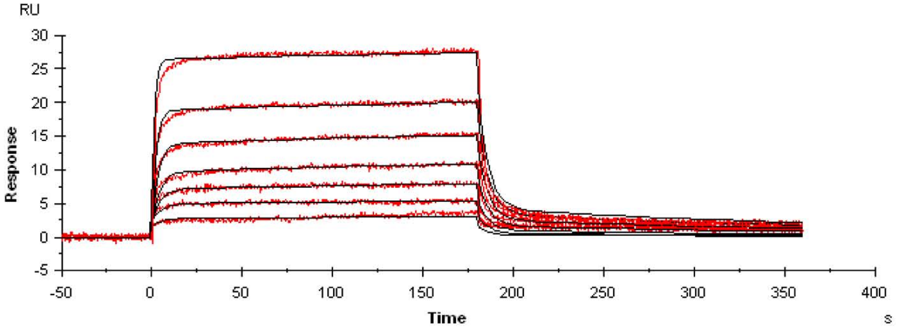

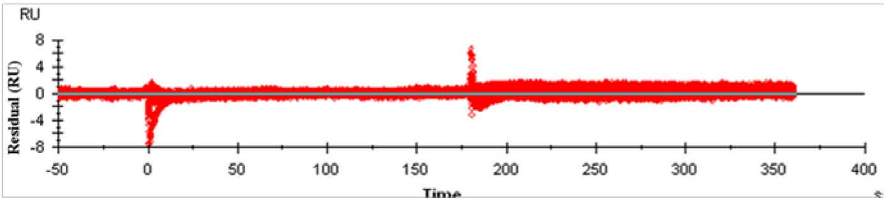

I) MTrP-DAP (amide/acid) (**6a)**

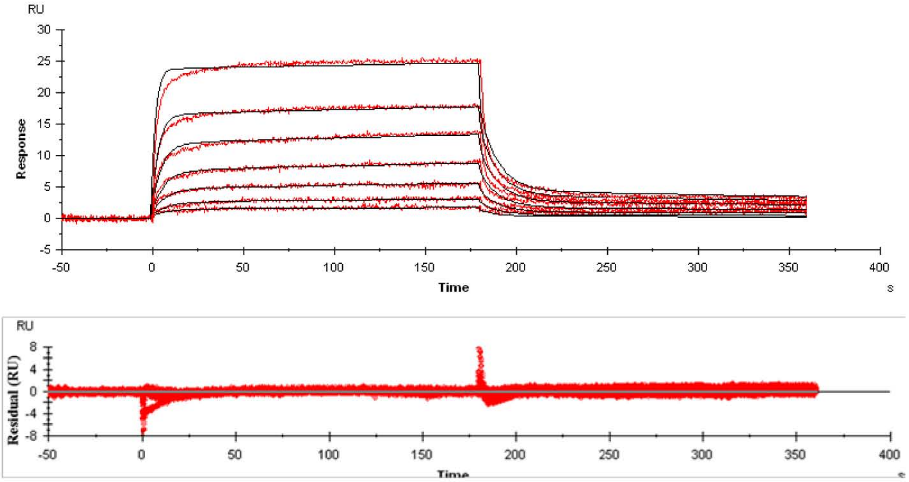

J) MTrP-DAP (acid/amide) (**6b)**

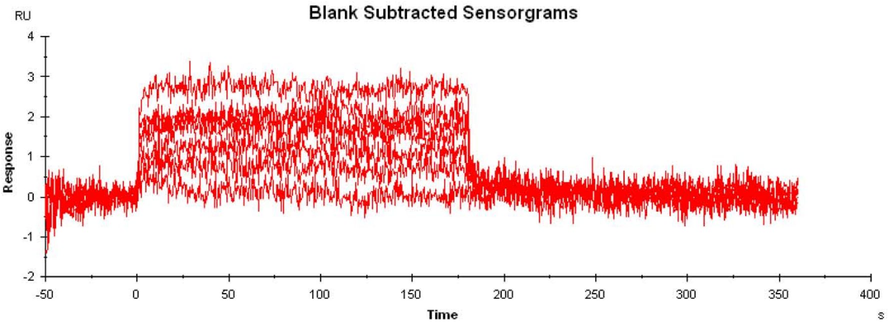

K) MTrP-DAP (acid/acid) (**6d)**

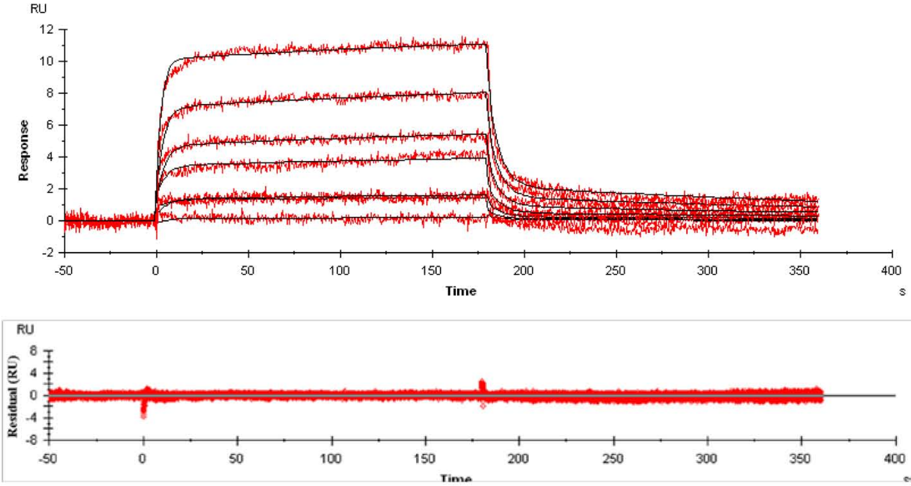

L) MTrP-DAP(amide/acid)NHAc **(6e)**

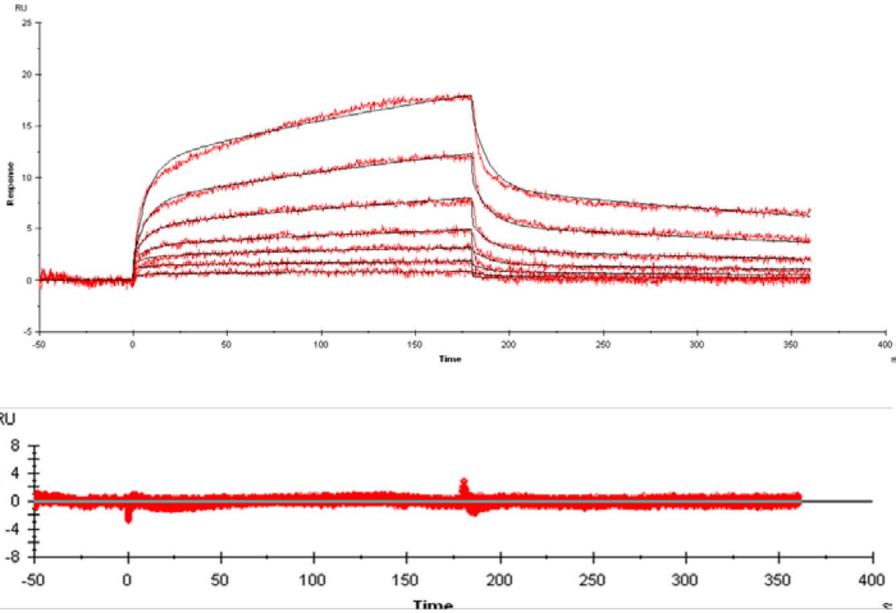

M) MPP-DAP (amide/acid) **(7)**

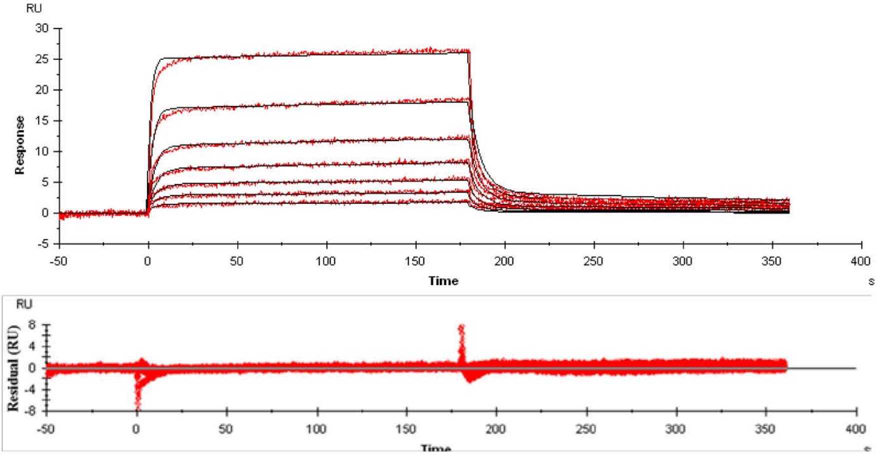

N) Peptide (**8**)

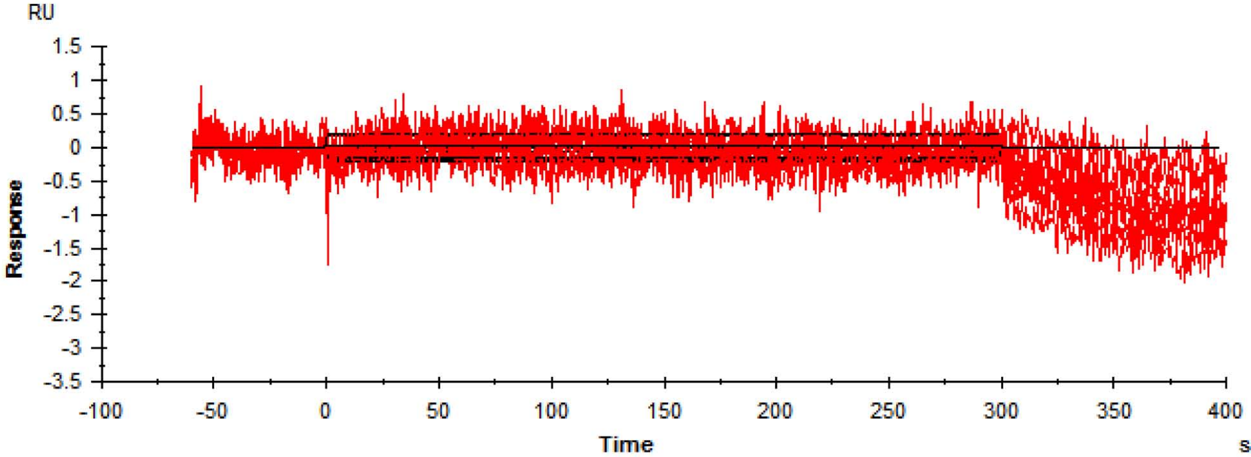

RU
1.5
1
0.5
0
-0.5
-1
-1.5
-2
-2.5
-3
-3.5
Response
-100
-50
0
50
100
150
200
250
300
350
400
Time
s
