# Supplementary material for: The Extracytoplasmic Domain of the Mycobacterium tuberculosis Ser/Thr Kinase PknB Binds Specific Muropeptides and Is Required for PknB Localization
Source: PLoS Pathog. 2011 Jul 28;7(7):e1002182. doi: 10.1371/journal.ppat.1002182 (PMC3145798; doi:10.1371/journal.ppat.1002182)
Supplement: Table S1 — Kinetic binding parameters for the interaction of synthetic muropeptides with the PASTA domains of M. tuberculosis PknB. (DOC) [file ppat.1002182.s006.doc]

**Table S1.** Kinetic binding parameters for the interaction of synthetic muropeptides with the PASTA domains of *M. tuberculosis* PknB

| Analyte | k_a1_  (10^-3^ s^-1^) | k_d1_  (10^-3^ s^-1^) | k_a2_  (10^-3^ s^-1^) | k_d2_  (10^-3^ s^-1^) | K_D_  µM) | χ^2^  (RU^2^) |
| --- | --- | --- | --- | --- | --- | --- |
| MTP-Lys (amide) **1** | - | - | - | - | >500 | - |
| MTrP-Lys (amide) **2a** | 82.93 | 16 | 44.5 | 5.5 | 21.5 | 0.6 |
| MTrP-Lys (amide) NHAc **2b** | - | - | - | - | >500 | - |
| MTrP-Lys (Gly) **2c** | - | - | - | - | > 500 | - |
| MPP-Lys (D-Ala) **3a** | - | - | - | - | > 500 | - |
| MPP-Lys (Gly) **3b** | - | - | - | - | >500 | - |
| Peptide **4** (amide) | - | - | - | - | > 500 | - |
| MTP-DAP (amide/acid) **5** | 5577--- | 172.9 | 1.64 | 3.86 | 21.8 | 0.3 |
| MTrP-DAP (amide/acid) **6a** | 3451 | 100.7 | 1.83 | 1.4 | 12.7 | 0.28 |
| MTrP-DAP (acid/amide) **6b** | - | - | - | - | >100 | - |
| MTrP-DAP (amide/amide) **6c** | 342.8 | 57.8 | 41.5 | 4.02 | 14.9 | 0.2 |
| MTrP-DAP (acid/acid) **6d** | 1931 | 168.7 | 2.0 | 3.2 | 53.6 | 0.08 |
| MTrP-DAP(amide/acid)NHAc **6e** | 354.2 | 106 | 6.6 | 2.2 | 73.8 | 0.09 |
| MPP-DAP (amide/acid) **7** | 4316 | 161.5 | 1.4 | 2.8 | 25.1 | 0.23 |
| Peptide **8** (amide/amide) | - | - | - | - | >500 | - |

The recombinant extracellular domain of PknB was immobilized on NHS-activated groups of a CM-5 sensor chip surface (5,000 RU) and titration experiments were performed with the synthetic compounds 1-8 (Figure 1). The binding constants of all compounds were determined by fitting the data using a two-state binding model. Sensorgrams for the kinetic analyses are presented in Figure S3. RU, resonance units; MTP, muramyl-tripeptide; MTrP, muramyl-tetra peptide; MPP, muramyl-pentapeptide.
