# Supplementary material for: The Extracytoplasmic Domain of the Mycobacterium tuberculosis Ser/Thr Kinase PknB Binds Specific Muropeptides and Is Required for PknB Localization
Source: PLoS Pathog. 2011 Jul 28;7(7):e1002182. doi: 10.1371/journal.ppat.1002182 (PMC3145798; doi:10.1371/journal.ppat.1002182)
Supplement: Protocol S1 — Methods for live cell imaging of M. smegmatis expressing RFP-PknB fusions. (DOCX) [file ppat.1002182.s007.docx]

Protocol S1

To determine the localization of PknB in live *M. smegmatis* cells, strains of *M. smegmatis* harboring fusions of *rfp* with full length or different domains of *pknB*, expressed under the acetamidase promoter, were grown in 7H9 to an O.D_600_ of 0.6-0.8. A 1% agarose pad containing 7H9 medium and 0.5% acetamide was prepared in a rectangular chamber made with Frame-Seal (Biorad) rectangular strips on a glass microscope slide. The *M. smegmatis* cells were passed through a 5μm pore filter and 3-4 μl of the culture was spotted onto the agarose pad. After spotting the cells, the chamber was sealed with a coverslip. The slide was incubated at 37^o^ C for 12 hrs and observed with a Nikon TE2000 microscope equipped with a 100x differential interference contrast (DIC) oil immersion objective and a red fluorescence filter. Images were captured by an OrcaER camera (Hamamatsu Photonics Inc.), acquired with SlideBook (Intelligent Imaging Innovations Inc.) and processed with Adobe Photoshop CS5.
